# Preparing for the Next Pandemic: Lessons from COVID-19’s Impact on Child and Adolescent Health Inequities in Ghana

**DOI:** 10.3390/bs15091187

**Published:** 2025-08-30

**Authors:** Franklin N. Glozah, Robert S. Tia

**Affiliations:** Department of Social and Behavioural Sciences, School of Public Health, University of Ghana, Accra 00233, Ghana

**Keywords:** COVID-19, child health, adolescent well-being, Ghana, inequalities

## Abstract

The pandemic spared most children and adolescents in Ghana from severe clinical disease, but it exposed long-standing gaps in services and protection methods. Methods: We conducted a desk-based narrative review of peer-reviewed studies, national and international reports, and grey literature from January 2020 to May 2025. The evidence was organised across eight domains of child and adolescent well-being. Across mental health, gambling and other risky behaviours, access to health services, economic hardship and child labour, nutrition, education, early childhood development, and WASH, the pandemic disrupted essential services and social safety nets. Examples include declines in routine care and immunisation, wider digital exclusion during remote learning, a rise in child labour linked to income loss, and persistent hygiene constraints. Preparedness in Ghana should focus on mental health, digital inclusion, early childhood services, and strong social protection. Ghana’s specific empirical data are uneven, so we triangulate peer-reviewed evidence with official reports, appraised the grey literature, and calibrated claims to the strength of sources.

## 1. Introduction

The COVID-19 pandemic, declared a Public Health Emergency of International Concern by the World Health Organization (WHO, 2020), unleashed a global crisis with disproportionate consequences for vulnerable populations, particularly in low- and middle-income countries (LMICs). While much of the early discourse focused on direct health outcomes, such as hospitalizations, morbidity, and mortality, emerging evidence now underscores the pandemic’s indirect and far-reaching impact on social determinants of health, especially among children and adolescents (UNICEF, 2021; Racine et al., 2021). In countries like Ghana, where child poverty, unequal access to services, and regional disparities already existed pre-pandemic, COVID-19 became a magnifier of long-standing inequities.

Although children and adolescents have been relatively spared from severe disease compared to adults (WHO, 2021), they bore the brunt of disruptions to education, healthcare access, mental health support, nutrition programmes, and early developmental services. These disruptions not only intensified pre-existing disparities, but also introduced new forms of vulnerability, particularly for children in low-income households, informal settlements, and underserved regions (WHO, 2021).

The crisis has also highlighted a critical weakness in Ghana’s public health infrastructure: its limited capacity to anticipate and respond to the unique needs of children and adolescents in emergencies. For example, education and health systems lacked contingency plans for service continuity during school closures or lockdowns, and most social protection programmes were not calibrated to address the age-specific risks faced by young people (WHO, 2020). As a result, many interventions were reactive, fragmented, and disproportionately adult-focused. The neglect of a child-centred preparedness lens has had significant developmental and psychosocial consequences, which may reverberate long after the pandemic subsides (UNICEF, 2021).

Ghana is a critical case because school closures ran from 16 March 2020 to 18 January 2021; routine service statistics are available through the District Health Information Management System 2 (DHIMS2); and National Health Insurance Scheme (NHIS) claims provide additional utilisation signals, alongside marked urban and rural contrasts (Malm et al., 2024).

Unlike previous narrative reviews, this paper situates child and adolescent outcomes within a public health preparedness lens, arguing that resilience-building must prioritize equity across essential child development systems.

After reviewing the literature, no Ghana-specific review addressing this topic across systems was found.

The objective of this paper is to synthesise Ghana-specific evidence on how COVID-19 affected children and adolescents across eight domains and to derive practical preparedness lessons for Ghana.

## 2. Materials and Methods

This review employed a comprehensive desk-based literature synthesis to examine the impact of the COVID-19 pandemic on health and well-being inequalities among children and adolescents in Ghana.

### 2.1. Data Sources and Search Strategy

This review drew on a wide range of data sources, including:

- Government policy documents and national reports, particularly those from the Ministry of Health, Ghana Health Service, and Ministry of Education;
- International organization reports, such as those from the World Health Organization (WHO), United Nations Children’s Fund (UNICEF), UNESCO, and the World Bank;
- Peer-reviewed journal articles and academic publications focused on public health, child development, mental health, education, and social determinants of health;
- Grey literature, including NGO reports, working papers, policy briefs, and media reports relevant to the Ghanaian context.

A structured search was conducted using databases such as PubMed, Scopus, Google Scholar, and institutional repositories. Keywords used in various combinations included COVID-19, children, adolescents, Ghana, inequalities, mental health, education, nutrition, WASH, economic hardship, and health system disruption.

### 2.2. Inclusion and Exclusion Criteria

Inclusion: studies focused on children and or adolescents in Ghana; examined direct or indirect effects of COVID-19; reported outcomes in health, education, nutrition, protection, or WASH; and published January 2020 to May 2025.

Exclusion: studies focused solely on adults; items not specific to Ghana; and non-empirical items without relevance to the outcomes.

### 2.3. Data Extraction and Thematic Synthesis

A data extraction matrix was developed to capture key details from each source, including publication type, author(s), year, thematic focus, population group, and main findings. Qualitative thematic analysis was used to identify recurring patterns across the literature. Two reviewers independently screened titles, abstracts, and full texts, and both extracted data into a shared matrix. A draft codebook was piloted on a random subset of sources and refined by consensus. Disagreements at any stage were resolved through discussion. Because the corpus mixed designs and grey literature, agreement was tracked and discussed rather than reduced to a single coefficient. We note this choice as a limitation.

### 2.4. Data Analysis

We conducted an evidence-weighted narrative synthesis. Steps included the familiarisation and open coding of extracted material, development and piloting of a codebook, double coding of a twenty-percent sample to check consistency, resolution of discrepancies by discussion, constant comparison to derive themes, and construction of a source by theme matrix. Heterogeneity of outcomes precluded meta-analysis.

### 2.5. Source Weighting and Quality Appraisal

To reflect the varied evidence base, we applied a pragmatic appraisal and weighting scheme. Peer-reviewed empirical studies conducted in Ghana were treated as the highest weight. Official statistics and reports from recognised agencies (for example, GSS, MOH, WHO, UNICEF, World Bank) were treated as high-quality administrative or programmatic evidence. Grey literature and media reports were used to provide context, triangulation, or timely signals, and were explicitly flagged as such. When findings diverged across source types, we prioritised peer-reviewed empirical work and official statistics, and we noted any uncertainty in the Results and Discussion Section.

### 2.6. Protocol Registration

This review was not registered in PROSPERO or a similar registry. The rationale is that it is a desk-based narrative synthesis without meta-analysis, drawing on both academic and grey sources that evolved rapidly during the pandemic. The full search strings and inclusion logic are now provided to enhance transparency, and we acknowledge non-registration as a limitation.

## 3. Results

We included 18 Ghana specific records across the eight domains, comprising 9 peer-reviewed empirical studies and 9 official or administrative sources. Table 1 summarises study-level characteristics, and Table 2 reports the number of Ghana-specific sources per domain with a brief description of sample coverage.

The findings are grouped into eight domains: mental health; gambling and risky behaviours; access to health services; economic hardship and child labour; nutrition; education; early childhood development; and WASH.

The review revealed that the COVID-19 pandemic has had a multifaceted impact on the lives of children and adolescents in Ghana, exacerbating existing inequalities and creating new challenges.

### 3.1. Mental Health: Disruptions, Stressors, and Service Gaps

COVID-19 triggered a substantial decline in mental well-being among children and adolescents globally, and Ghana was no exception (Ahorsu et al., 2020; Abu-Baidoo, 2022). Prolonged school closures, social isolation, exposure to familial stress, and uncertainty about the future combined to produce elevated levels of anxiety, depression, and behavioural distress among youths (Ford et al., 2021; Racine et al., 2021). In Ghana, national lockdowns and restrictions on movement disrupted children’s routines and cut off vital social networks, including teachers, peers, and community mentors—networks that often serve as protective factors in adolescent psychosocial development.

Emerging studies reveal that the psychological toll was disproportionately felt by children from socioeconomically disadvantaged backgrounds, who not only faced emotional isolation, but also lived in overcrowded and often unstable home environments (GSS, 2020a; Owusu & Frimpong-Manso, 2020). For adolescents, added responsibilities, including economic contributions to the household and caring for younger siblings, compounded stress levels, while stigma related to COVID-19 exposure or infection added further psychological strain (GSS, 2020b).

Mental health services, already limited pre-pandemic, were largely inaccessible during lockdown periods. Ghana has a severe shortage of child and adolescent mental health professionals, with just a handful of psychologists and social workers allocated to this age group nationally (WHO, 2022). Although mental health support was briefly integrated into some radio and community-based health programmes, these were neither widespread nor targeted to adolescent-specific needs. Furthermore, no nationwide psychological first aid or trauma-informed interventions were rolled out for children affected by loss or grief during the pandemic.

Even before the pandemic, Ghana faced a serous, long-standing mental health treatment gap: between 94% and 99% of individuals with probable mental and neurological conditions went undetected in rural primary care settings (Ae-Ngibise et al., 2023) The onset of COVID-19 likely worsened this gap. Globally, depression and anxiety increased by more than 25% in the first year of the pandemic, with 63% of young people reporting significant symptoms (WHO, 2021, 2022). Given Ghana’s severe shortage of child and adolescent mental health services, these increases in psychological distress would have been largely unmitigated.

The lack of integration between mental health and school systems further undermined resilience. Globally, countries that maintained psychosocial service continuity through schools (e.g., Canada, New Zealand) protected youth from long-term harm (Cost et al., 2022). Ghana’s lack of remote school-based mental health services during school closures thus widened the gap in emotional support provision.

COVID-19 exposed and intensified the fragility of Ghana’s child and adolescent mental health infrastructure. The psychosocial toll was not merely a function of the virus, but of the system’s failure to anticipate and buffer emotional distress during a public health emergency. A preparedness agenda for future pandemics must prioritize mental health system integration, including scalable school-based psychosocial services, mobile counselling initiatives, and youth-specific mental health literacy campaigns.

### 3.2. Gambling and Addictive Behaviours: A Silent Risk Amplified by Isolation and Invisibility

While often overlooked in child and adolescent health discussions, gambling emerged as a significant and under-acknowledged public health concern during the COVID-19 pandemic. As restrictions on movement, curfews, and school closures took effect, many adolescents in Ghana, especially those already socially or economically marginalised, turned to online gambling platforms in increasing numbers (Glozah et al., 2021). The closure of land-based gambling venues and suspension of organised sports created a vacuum that was quickly filled by digital betting, particularly via mobile sports platforms and virtual casino apps.

Ghana’s youth gambling landscape is shaped by both accessibility and cultural permissiveness. Although gambling is legally restricted for individuals under 18 years old, enforcement remains weak, and the proliferation of smartphones and mobile money platforms has made gambling both accessible and anonymous for underage users (Abbott, 2020). With traditional sources of entertainment and social interaction curtailed during the pandemic, gambling became a readily available, low-barrier form of engagement and escape.

The evidence from global systematic reviews suggests that adolescents are particularly vulnerable to gambling-related harm due to developmental impulsivity, peer influence, and limited risk perception (Gainsbury et al., 2022). In Ghana, the situation was further exacerbated by poor digital regulation, weak parental monitoring, especially in overcrowded households—and aggressive advertising by gambling companies during televised COVID-19 relief programmes and football reruns. Anecdotal reports and small-scale surveys conducted in Accra and Kumasi suggest a spike in sports betting among adolescents aged 15–19 years in 2020–2021, with many reporting financial losses, anxiety, and addictive tendencies (Human Rights Watch, 2021a; Hodgins & Stevens, 2021). Nationally, a 2022 study of 5024 children aged 8–17 years found 3.1% had engaged in gambling activities. (Kyei-Gyamfi et al., 2022).

Beyond financial consequences, adolescent gambling during the pandemic was linked to increased school absenteeism, familial conflict, and exposure to illicit lending networks. These risks disproportionately affected boys, though emerging patterns suggest a rise in female adolescent gambling via social casino games. Crucially, Ghana lacks a dedicated adolescent addiction prevention strategy, and youth-specific services for gambling disorders are virtually non-existent.

In rural areas, as early as pre-pandemic times, up to 40% of male and 30% of female adolescents reported a gambling problem in the prior year (Odame et al., 2021). Fast forward to the pandemic-era data from the Volta Region, a striking 84% of youths gambled, with 40% classified as problematic gamblers (Manu et al., 2024). Among these gamblers, 43.6% experienced depression, 68.8% experienced anxiety, and 31.1% reported stress, underscoring a dramatic escalation in both prevalence and psychological harm (Manu et al., 2024).

The pandemic amplified a silent epidemic of adolescent gambling, revealing a blind spot in Ghana’s public health and child protection systems. While gambling may seem a secondary concern amidst more visible challenges, its developmental, social, and economic consequences are deeply entwined with broader inequalities. Preparedness strategies must now include digital risk governance, targeted education campaigns on gambling harms, and integration of behavioural addiction screening into adolescent health services, especially in school and community settings.

### 3.3. Health System Disruptions: A Fragile System Under Strain—Implications for Adolescent Health Equity

Ghana’s health system, while making gradual progress in maternal and child health over the past two decades, entered the COVID-19 pandemic with significant structural limitations, particularly in service integration, workforce capacity, and adolescent-responsive care. The outbreak sharply exposed and magnified these weaknesses, with devastating implications for access to essential health services among children and adolescents (WHO, 2020; Church et al., 2020).

During the pandemic’s peak, healthcare resources were reallocated toward emergency COVID-19 response activities, testing, case management, isolation centres, and contact tracing. This diversion disrupted routine services, including immunisation, growth monitoring, sexual and reproductive healthcare, and adolescent counselling services (Dwomoh et al., 2023; UNFPA, 2020). Many facilities reported stockouts of vaccines, contraceptives, and micronutrients, with outreach services suspended in several districts due to movement restrictions and fear of community transmission.

Adolescents, particularly girls, faced unique barriers. Sexual and reproductive health (SRH) services, including access to contraceptives, emergency contraception, and safe abortion care, became difficult to access, especially in rural and peri-urban settings. Reports suggest increased rates of unintended pregnancies and sexually transmitted infections among adolescent girls in 2020–2021, exacerbated by limited service availability and stigma around seeking SRH care (UNFPA, 2020; Church et al., 2020). These trends mirror global findings that show a rollback in SRH access across LMICs during the pandemic (Roberton et al., 2020).

Moreover, health-seeking behaviour was significantly affected by misinformation, stigma, and a fear of infection at healthcare facilities. Many parents delayed or avoided seeking care for their children, unless critically necessary (WHO, 2021). The situation was worsened by the lack of clear communication strategies targeting youth and caregivers about the availability of non-COVID-19 services.

While other countries implemented mobile clinics, telehealth solutions, and community outreach programmes to sustain service continuity, Ghana’s adoption of such innovations remained limited. Although telemedicine was piloted in a few urban facilities, poor digital infrastructure, low internet penetration, and lack of adolescent-specific adaptation prevented meaningful uptake (Marie Stopes UK, 2020). No large-scale strategy emerged to ensure continuity of care for adolescents during lockdown.

Quantitatively, the magnitude of disruption was non-trivial. Using nationwide DHIMS2 data across all 260 districts, routine child immunization doses fell by ~6% in April 2020 (vs. April 2019), with a larger mean drop of ~14% in the 40 lockdown-affected districts; services broadly recovered to pre-pandemic growth by 2021 (Durizzo et al., 2024). Complementing this, the WHO reports that DTP3 coverage declined from 99% (2019) to 97% (2020) in Ghana, leaving >32,000 children without full protection (WHO AFRO, 2023). Claims-based interrupted time-series covering 502 facilities show month-on-month declines of ~21,948 antenatal care (ANC) visits and ~151,342 outpatient (OPD) visits after restrictions began, while postnatal and delivery volumes changed little overall (Opoku-Boateng et al., 2024). District case studies further documented reduced ANC/PNC attendance and temporary halts to routine childhood vaccinations during lockdown (Koka et al., 2024).

The COVID-19 crisis highlighted Ghana’s limited preparedness for safeguarding essential adolescent health services during emergencies. Disruptions in SRH, immunisation, and preventive care reversed hard-won gains and widened existing disparities. Moving forward, health system strengthening must include adolescent-responsive planning, service continuity protocols, and investment in alternative delivery mechanisms, such as mobile health and community outreach, especially for underserved populations.

### 3.4. Economic Insecurity and Child Labour: Poverty, Precarity, and the Erosion of Childhood

The economic fallout from COVID-19 triggered widespread financial distress in Ghana, exacerbating child poverty and pushing thousands of families into survival mode. Lockdowns, border closures, and disruptions to the informal sector, which accounts for over 70% of Ghana’s employment, led to business closures, job losses, and sharp reductions in household income, particularly in urban poor and peri-urban communities (GSS, 2020b; Soliku et al., 2021). For many families, especially female-headed households, children were rapidly absorbed into the economic coping equation. National phone-survey data show that 77.4% of households reported a decrease in total income since 16 March 2020, with 52.1% reducing food consumption as a coping strategy (GSS, 2020a).

The pandemic saw a visible resurgence of child labour, particularly in sectors such as street hawking, fishing, petty trade, and small-scale mining. Human Rights Watch (2021b) documented numerous cases of children in Accra and Cape Coast dropping out of school to work as food vendors or load carriers, often working long hours under hazardous conditions. Monitoring data from June 2020 indicate a 10.9% increase in the share of children aged 5–17 years engaged in working or selling activities in the previous 30 days, compared with the pre-pandemic period before 16 March 2020 (UNICEF Ghana et al., 2021a). Adolescents, in particular, were drawn into informal work, motorbike deliveries, construction support, and cleaning, often for exploitative wages and without any form of protection or oversight.

This crisis was not merely economic but developmental. Engaging in labour during critical educational and developmental periods compromises not only academic outcomes but long-term health and psychosocial well-being (ILO, 2021). Girls faced a dual burden: participating in income-generating activities while shouldering increased domestic responsibilities, such as caregiving for younger siblings when schools were closed. The long-term consequences of this regression are profound; interruptions in schooling heightened vulnerability to exploitation and the erosion of pathways out of poverty.

While the Government of Ghana launched several emergency cash transfer and food distribution initiatives, including expansions to the Livelihood Empowerment Against Poverty (LEAP) programme, coverage was limited, and child-specific vulnerabilities were not explicitly addressed (UNICEF, 2021). These programmes, though laudable, were not calibrated to prevent school dropouts, child labour, or support adolescent girls at risk of early marriage or exploitation. There was also a notable absence of monitoring mechanisms to assess how children within households were affected by these economic shocks.

COVID-19 laid bare the fragility of child protection and social welfare systems in Ghana. Economic stress translated directly into child vulnerability, undoing years of progress on child labour reduction. Future pandemic preparedness must integrate child-sensitive social protection schemes with built-in shock responsiveness, adolescent-specific targeting, and monitoring tools to prevent the normalization of child labour during crises.

### 3.5. Nutrition and Food Security: When Plates Empty: Malnutrition as an Invisible Pandemic

The COVID-19 pandemic disrupted food systems globally, but for children and adolescents in Ghana, many of whom rely on school feeding programmes or already live in food-insecure households, the impact was immediate and profound. School closures in particular severed access to daily meals for thousands of children, exposing the critical, and often overlooked, role of schools as nutrition delivery platforms (FAO et al., 2021). Pre-pandemic, Ghana’s School Feeding Programme served about 2.8 million pupils; by September 2020, 89.4% of households with children aged 6–14 years who usually received school meals reported receiving none in the prior four weeks (Botchwey, 2021; UNICEF Ghana et al., 2021b). For vulnerable households, particularly in northern Ghana and urban slums, this disruption compounded pre-existing nutritional deficits.

Reports from the Ghana School Feeding Programme and UNICEF Ghana (2020) revealed significant increases in household food insecurity in 2020–2021, driven by inflation, loss of income, and market supply chain interruptions. National FIES estimates indicate 47.7% of the population experienced moderate or severe food insecurity in June 2020 (including 9.5% severe) and 47.0% (with 6.2% severe) in September 2020 (GSS, 2021). The consequences were particularly severe for children under five years old and adolescents undergoing rapid physical and cognitive development. These groups experienced rising rates of undernutrition, micronutrient deficiencies, and in some cases, acute malnutrition. Anecdotal field reports noted reductions in dietary diversity and increased consumption of calorie-dense but nutrient-poor foods, often the only affordable option in constrained household budgets (Headey et al., 2020). UNICEF noted that by September 2020, food insecurity remained high, threatening nutrition in roughly one in five children aged 6 months–14 years (UNICEF Ghana et al., 2021a).

Ghana’s nutrition challenges during the pandemic were not solely a result of economic hardship. The health system’s redirection toward pandemic response led to a widespread disruption in community-based growth monitoring, maternal and child nutrition counselling, and micronutrient supplementation services (WHO, 2021). Outreach activities by community health nurses were curtailed, resulting in missed opportunities for early detection and management of malnutrition. Additionally, Ghana’s nutrition surveillance mechanisms, already fragmented prior to the pandemic, were insufficiently responsive to capture rapid shifts in food insecurity at the community level (Laborde et al., 2021).

Global projections have painted a dire picture of the long-term consequences of pandemic-era undernutrition. The Lancet Global Health modelling study estimated that COVID-19-related service disruptions could lead to an additional 6.7 million children suffering from wasting in 2020 alone, primarily in LMICs (Roberton et al., 2020). In Ghana, although comprehensive national data are still emerging, district-level reports suggest a rise in childhood stunting and wasting, especially in regions already facing high malnutrition rates pre-pandemic.

COVID-19 starkly revealed the fragility of child nutrition systems in Ghana, particularly in how dependent they are on schools, informal economies, and overstretched health workers. Building resilience against future shocks requires integrated strategies that combine household-level food security interventions with decentralized nutrition surveillance and robust school-based feeding systems. Nutrition must be viewed not only as a health outcome but as a foundational pillar of child survival, development, and pandemic preparedness.

### 3.6. Education: Learning Loss, Digital Exclusion, and the Deepening Divide

Among the most immediate and visible effects of the COVID-19 pandemic was the unprecedented disruption to education systems. In Ghana, all educational institutions were closed in March 2020, affecting approximately 9.2 million learners across basic and secondary levels (UNESCO, 2020). Although necessary for public health containment, these closures inadvertently amplified pre-existing educational inequities, particularly for children from low-income families, rural communities, and marginalized groups.

The transition to remote learning exposed stark digital divides. While some urban private schools quickly shifted to online platforms, the vast majority of public school students were left behind due to a lack of access to devices, internet connectivity, or even reliable electricity (Owusu & Frimpong-Manso, 2020). Although the Ghana Education Service (GES), in partnership with the Ministry of Education, implemented radio and television-based instruction, participation was uneven, and many students reported low engagement and a difficulty understanding the content without teacher interaction or parental support.

By September 2020, a nationally representative phone survey showed that among households with children in primary and junior high school, 43.3% in urban areas versus 33.0% in rural areas reported watching educational television, while listening to educational radio was 26.5% in rural areas versus 6.1% in urban areas; among senior high-school learners, use of mobile learning apps and e-learning reached 26.2% and 9.2% in urban households compared with 5.5% and 4.3% in rural households (UNICEF Ghana et al., 2021b).

Girls were disproportionately affected. The closure of schools increased the risk of early pregnancy, domestic responsibilities, and school dropout, especially in regions already grappling with gender disparities in education. Evidence from the United Nations Population Fund (UNFPA, 2020) and World Bank (2021) suggests that adolescent girls in LMICs, including Ghana, are more likely to disengage permanently from schooling due to household economic pressures, caregiving roles, or gender norms that deprioritize their education during crises.

Learning loss was not only academic, but developmental. Beyond formal instruction, schools provide structure, social interaction, and protection for vulnerable children. The suspension of school-based counselling, peer support programmes, and extracurricular activities undermined the broader developmental ecosystem that supports psychosocial well-being and resilience.

Furthermore, there was little provision for learners with disabilities, many of whom were excluded from mainstream online or broadcast learning options. This widened educational exclusion and underlined the absence of an inclusive education preparedness framework. Even when schools reopened in 2021, safety protocols, resource gaps, and absenteeism continued to hinder learning recovery, particularly in public schools serving low-income communities (World Bank, 2021).

Nationally representative data collected in March 2021 indicate that while dropout remained about 2 percent, grade repetition increased from roughly 3.5 percent in 2018 to 10.5 percent post-pandemic, with a higher risk among poorer children (IEPA & Center for Global Development, 2022).

The educational fallout from COVID-19 in Ghana reflects a system unprepared for a crisis, especially in delivering equitable and inclusive learning. Future preparedness must prioritize digital equity, targeted learning recovery, inclusive education, and the integration of education into broader social protection planning. Investment in education technology infrastructure, teacher digital training, and resilient hybrid models is critical, not just for continuity in emergencies, but for equity in all contexts.

### 3.7. Early Childhood Development (ECD): An Important Stage Interrupted

Early childhood is a period of rapid brain development and foundational learning, deeply sensitive to environmental stimuli, caregiver interaction, and consistent access to health, nutrition, and play-based education (Center on the Developing Child at Harvard University, 2017). The COVID-19 pandemic disrupted these essential conditions in Ghana, threatening the cognitive, emotional, and physical development of children in their most formative years.

With the closure of preschools, kindergartens, and early childhood centres, many young children, particularly those from low-income families, were abruptly disconnected from structured learning, safe play spaces, and socialization opportunities. The Ghana Education Service did not extend remote learning programmes to children below primary school level, reflecting a global trend where early learners were deprioritized in emergency education responses (UNICEF, 2021; Yoshikawa et al., 2020). For these children, the absence of early stimulation, social engagement, and routine may have long-lasting developmental consequences.

Compounding the problem was the increased economic and psychological stress experienced by caregivers during the pandemic. Studies show that parental distress, food insecurity, and crowded living conditions negatively affect caregiving quality, reducing the availability of responsive interactions critical for early learning and emotional security (Kagan, 2021; Ford et al., 2021). In Ghana, such stressors were particularly acute in urban informal settlements and among families already struggling with poverty prior to the pandemic.

Health and nutrition services for young children were similarly disrupted. Many routine growth monitoring activities, vitamin A supplementation, and home visits by community health nurses were suspended or scaled back during lockdown periods (WHO, 2020). As a result, opportunities for the early identification of developmental delays or malnutrition were missed, particularly in rural and underserved areas.

Despite the known long-term return on investment in early childhood development, Ghana’s national COVID-19 response largely overlooked this age group. Unlike countries such as Kenya and Rwanda, which issued targeted policy guidance on protecting ECD services during lockdown, Ghana lacked a coordinated intersectoral strategy for mitigating developmental risk in children under five years old (World Bank, 2021).

The interruption of early childhood development services during COVID-19 represents a silent crisis with potentially lifelong consequences. To prepare for future public health emergencies, Ghana must recognize early childhood as a core pillar of resilience and social equity. This will require integrating ECD into national preparedness and response plans, strengthening parenting support programmes, and investing in community-based ECD services that are accessible, adaptable, and inclusive.

Ghana’s pre-primary and basic schools were closed nationwide from 16 March 2020 until 18 January 2021—roughly 10 months—disrupting early childhood services for an estimated 9.2 million KG-SHS learners (plus ~0.5 million tertiary students) (GES, 2020, 2021a; World Bank, 2022). During closures, children who had previously attended higher-quality ECE showed higher remote-learning engagement (+0.14 SD in October 2020), suggesting that quality ECE buffered some learning disruptions (S. Wolf et al., 2022). Consistent with the prolonged shutdown, most ECE centres remained closed in 2020, and preschool teachers reported income loss and operational strain (Obeng et al., 2022).

### 3.8. Water, Sanitation, and Hygiene (WASH): Hygiene Inequities in a Time of Contagion

The COVID-19 pandemic underscored the vital role of water, sanitation, and hygiene (WASH) in disease prevention, yet it simultaneously revealed and exacerbated longstanding disparities in WASH access for children and adolescents in Ghana. In the face of a virus that demanded frequent handwashing and safe distancing, many children, particularly those living in informal urban settlements and underserved rural areas, were left without the basic means to protect themselves (WHO/UNICEF JMP, 2021). Even before COVID-19, while more than half of households had a designated place for handwashing, only about one in five had both water and a cleansing agent available at home (UNICEF Ghana, 2024).

Prior to the pandemic, over 25% of Ghanaian households lacked access to safely managed drinking water, and approximately 43% had no access to basic handwashing facilities with soap and water (GSS, 2018). The implementation of preventive measures, such as regular handwashing and surface sanitation, proved impractical in many low-income communities, schools, and childcare facilities due to the absence of infrastructure, a consistent water supply, and hygiene products (J. Wolf et al., 2021).

By 2021, the WHO/UNICEF JMP estimated that less than half of urban residents (46.5%) used basic hygiene services (a handwashing facility with soap and water at home), while 62.6% of urban residents used safely managed drinking-water services—underscoring persistent service gaps (WHO/UNICEF JMP, 2021).

Children in overcrowded households, especially in urban slums like Nima and Agbogbloshie, were disproportionately exposed to heightened infection risks due to shared sanitation facilities, an inability to maintain physical distancing, and poor ventilation. Public handwashing stations installed during the early months of the pandemic were often short-lived, poorly maintained, or in locations inaccessible to children, particularly those with disabilities or those living in remote areas (UNICEF Ghana, 2020).

Consistent with these structural constraints, a national-scale online survey during COVID-19 found only 48.4% of respondents adhered to good handwashing practices, despite good knowledge and positive attitudes (Omari et al., 2022).

In schools, WASH deficiencies hindered safe reopening and learning continuity. Many basic schools lacked functioning latrines, running water, or hand hygiene stations, contravening reopening safety protocols issued by the Ghana Education Service. The result was sporadic attendance, school closures due to local outbreaks, and reduced confidence among parents in sending children back to class, particularly girls, who are disproportionately affected by inadequate sanitation during menstruation (WHO/UNICEF JMP, 2021). When schools reopened in January 2021, GES required on-site handwashing with soap and running water (or alcohol-based sanitiser) as part of enhanced hygiene protocols (GES, 2021b).

Moreover, the pandemic response missed an opportunity to leverage the crisis to accelerate investment in child-friendly WASH systems. While emergency WASH campaigns promoted hand hygiene, few were tailored to children, integrated into school curriculums, or sustained beyond initial donor-funded efforts (WHO & UNICEF, 2022).

COVID-19 made clear that WASH is not just a public health measure; it is a child rights and equity issue. Ghana’s preparedness for future pandemics must embed WASH within health, education, and social protection strategies. This includes scaling up investment in child-accessible WASH infrastructure in homes, schools, and public spaces, as well as promoting hygiene literacy among children through culturally relevant materials and sustained practice in schools and communities.

## 4. Discussion

The objective of this paper was to synthesise Ghana specific evidence on how COVID-19 affected children and adolescents across eight domains and to derive practical preparedness lessons for Ghana.

The findings point to the fact that the COVID-19 pandemic exposed and amplified pre-existing structural inequities in Ghana’s child and adolescent health ecosystem. While children were less susceptible to the direct clinical impacts of the virus (WHO, 2021), they endured profound and disproportionate disruptions to the social and service systems that underpin their development. Across diverse domains, mental health, education, nutrition, and child protection, the evidence points to a pandemic that functioned as both a magnifier and multiplier of systemic disadvantage (UNICEF, 2021; Racine et al., 2021; Azevedo et al., 2020).

A key finding from this review is the cascading nature of vulnerability. Economic shocks triggered by job losses and informal sector disruptions (GSS, 2020a) resulted in increased household poverty, which in turn heightened food insecurity (FAO et al., 2021), pushed children into exploitative labour (ILO, 2021), and decreased school re-enrolment, especially among adolescent girls (UNFPA, 2020; World Bank, 2021). These effects were not isolated but syndemic, interacting in ways that compounded harm and intensified the burden on already marginalized populations (Singer et al., 2017; Roberton et al., 2020).

For mental health, the psychosocial toll was intensified by service inaccessibility and a lack of tailored interventions. Studies across sub-Saharan Africa confirm significant increases in depression, anxiety, and emotional distress among adolescents during the pandemic, with limited avenues for diagnosis or care (Racine et al., 2021; Ford et al., 2021; Abu-Baidoo, 2022). While some high-income countries responded with remote counselling and telepsychiatry, Ghana’s infrastructure limitations and absence of adolescent-targeted psychosocial policy left most young people unsupported (Cost et al., 2022).

Education disruptions were similarly stratified. While affluent learners transitioned to digital platforms, the vast majority of Ghanaian children, particularly those in rural areas or public schools, had little or no access to remote learning (Owusu & Frimpong-Manso, 2020; UNICEF, 2021). This digital divide reflects broader patterns seen across the Global South, where less than 50% of households have internet access (UNESCO, 2020). Learning losses were most acute among early learners and those with disabilities, with projections indicating that pandemic-era learning deficits may reduce lifetime earnings and future productivity (Azevedo et al., 2020; World Bank, 2021).

Nutrition outcomes followed a similarly concerning trajectory. School closures disrupted school feeding programmes, a vital source of daily nutrition for many children. Combined with price shocks and declining household incomes, these disruptions worsened child hunger, dietary monotony, and malnutrition (Headey et al., 2020; Laborde et al., 2021). During the COVID-19 pandemic, Ghana experienced a severe interruption of its school feeding programme: more than 2.9 million children who had been entitled to school meals missed out on them for up to ten months, undermining their nutrition, concentration, and ability to engage in learning—even as the government tried to focus support on JHS 3 and SHS 3 students preparing for final examinations (UNICEF Ghana & World Food Programme, 2021).

From a systems perspective, the pandemic revealed a lack of preparedness in integrating child- and adolescent-specific needs into national emergency frameworks. Many national taskforces lacked education and child development representation. Health system guidelines did not prioritize continuity of sexual and reproductive health (Church et al., 2020), nor did social protection responses distinguish child-specific vulnerabilities (Human Rights Watch, 2021b; UNICEF Ghana, 2020). This institutional blind spot echoes the findings from other LMICs, where pandemic plans were largely adult-centric (Guglielmi et al., 2020; Yoshikawa et al., 2020).

Global examples illustrate that better outcomes are possible. In Uruguay, child protection workers were rapidly redeployed to monitor at-risk households; in Finland, telehealth access was expanded to youths; and in Rwanda, educational radio was delivered in local languages with inclusive content (UNICEF, 2021; Cost et al., 2022). Ghana’s experience, while reflective of many LMICs, illustrates the urgent need to embed a rights-based, equity-focused approach into all aspects of preparedness and response.

In sum, COVID-19 was not just a health crisis, it was a development shock with generational consequences. The absence of child-centred preparedness mechanisms magnified existing disparities, revealing that resilience cannot be built on adult-focused planning alone.

Preparedness should embed child and adolescent needs across routine systems. In health, they should expand the child and youth mental health workforce and integrate brief psychosocial support into primary care and school health. In education, the School Health Education Programme should be resourced to deliver basic counselling, referral, and digital learning support, and protect school feeding during shocks. In social protection, cash transfers should be shock-responsive with adolescent-sensitive criteria, and the risks of child labour should be monitored in real time. In WASH, ring fence funds should be made available for child-accessible water and sanitation in schools and public spaces, with local maintenance plans. Across sectors, data systems that disaggregate by age, sex, disability, and region should be strengthened so that early warnings trigger rapid adjustments. These are feasible within existing mandates of GHS, GES, MoGCSP, and MMDAs, and align with the WHO and UNICEF guidance cited in this review.

### Limitations

Ghana-specific empirical data were uneven in several domains, so we triangulated peer-reviewed studies with official statistics and carefully appraised the grey literature. We therefore avoid causal attribution and calibrate the strength of claims to the quality of sources. Heterogeneity of outcomes and measures precluded meta-analysis. Publication and reporting bias may have influenced the available evidence. Also, the review draws on a mix of peer-reviewed studies, agency reports, and grey literature. While this improves coverage during a fast-moving emergency, it introduces variability in study quality and measurement. We mitigated this through a transparent weighting scheme and by privileging peer-reviewed Ghana-based evidence and official statistics where available. The review was not registered in PROSPERO, which we note could increase the risk of selective reporting; we addressed this by detailing our search strategy, inclusion criteria, and data handling.

## 5. Conclusions

The COVID-19 pandemic has served as a stress test for Ghana’s commitment to child and adolescent health equity. It revealed that while the biomedical impacts of the virus were less severe in younger populations, the broader societal, educational, and economic consequences were profoundly destabilizing. Children and adolescents were not spared—they were sidelined.

This review has shown how pre-existing inequalities were magnified across multiple, interconnected domains. From disrupted learning and rising child labour to gaps in mental healthcare and deteriorating nutrition, the crisis exposed both the vulnerabilities of youth and the systemic gaps in national preparedness. The lack of adolescent-centred planning, service integration, and inclusive surveillance left many young people without the support, protection, and continuity they needed during one of the most destabilizing periods in recent history.

To prepare for the next pandemic, or any future emergency, Ghana must reposition children and adolescents at the centre of its public health and development strategies. This requires more than policy statements; it demands sustained investment in child-responsive systems, cross-sector coordination, inclusive data collection, and crisis response protocols that recognize young people not as passive recipients of aid, but as a priority population with distinct needs and rights.

Preparedness is not merely about anticipating disease. It is about protecting human potential. For Ghana, the road to resilience must begin with its youngest citizens.

## Figures and Tables

**Table 1:** Ghana-specific sources included in the results by domain, with study characteristics and sample coverage.

Domain	First Author, Year	Source Type	Design or Data Source	Population or Setting	Sample Coverage or Frame	Key Outcome Used in Text
Mental health	(Weobong et al., 2023)	Peer reviewed	Situation analysis of district services	Five districts in Ghana	Service availability and treatment gap	Very high treatment gap in Ghana
Gambling and risky behaviours	(Odame et al., 2021)	Peer reviewed	Cross-sectional survey	Rural adolescents in Ghana	Adolescent respondents in rural communities	Gambling problem prevalence by sex
Gambling and risky behaviours	(Kyei-Gyamfi et al., 2022)	Peer reviewed	National cross-sectional survey	Children 8 to 17 years	n = 5024	Gambling history, type of gambling
Health system access	(Durizzo et al., 2024)	Peer reviewed	DHIMS2 analysis	All 260 districts	Routine doses month by month	Change in routine immunisation in 2020
Health system access	(Opoku-Boateng et al., 2024)	Peer reviewed	Interrupted time series using NHIS claims	**502** facilities nationwide	Monthly volumes in ANC, OPD, PNC, deliveries	Change in service volumes after restrictions
Health system access	(Koka et al., 2024)	Peer reviewed	Two district case study	Urban and rural districts	Facility records and interviews	Disruption to ANC, PNC, child vaccination
Health system access	(WHO AFRO, 2023)	Official report	Programme reports	National	DTP3 coverage 2019 to 2020	Decline in DTP3 coverage
Economic hardship and child labour	(GSS, 2020a)	Official survey brief	Households and Jobs Tracker, Wave 1	National households	National phone survey	Income loss and food related coping
Economic hardship and child labour	(UNICEF Ghana et al., 2021a)	Official report	National monitoring	Children and households in Ghana	Monthly snapshots through 2020	Increase in children working or selling
Nutrition	(Botchwey, 2021)	Peer reviewed	Policy and programme analysis	Ghana School Feeding Programme	About 2.8 million pupils pre-pandemic	School meals availability during closures
Nutrition	(GSS, 2021)	Official survey brief	Food Insecurity Experience Scale	National population	June and September 2020 estimates	Moderate and severe food insecurity
Nutrition	(UNICEF Ghana et al., 2021b)	Official report	National monitoring	Children and households in Ghana	September 2020 wave	Loss of school meals, nutrition risk
Education	(UNICEF Ghana et al., 2021a)	Official report	National phone survey	Households with learners	Urban and rural participation by modality	Remote learning engagement by TV, radio, apps
Education	(GES, 2020)	Official plan	Education response plan	National system	National policy and implementation	Closure timing and remote provision
Education	(GES, 2021b)	Official guidance	Re opening guidelines	Schools in Ghana	National policy	Hygiene and reopening protocols
Education	(World Bank, 2022)	Official results brief	Country case brief	Learners in Ghana	About 9.2 million KG to SHS learners	Duration of closures and implementation
Early childhood development	(S. Wolf et al., 2022)	Peer reviewed	Follow-up analysis	Children with prior ECE exposure	Change in remote engagement, SD +0.14	Protective effect of higher quality ECE
Early childhood development	(Obeng et al., 2022)	Peer reviewed	Qualitative study	ECE teachers and centres	National sample of preschools	Closures and teacher income loss
WASH	(Omari et al., 2022)	Peer reviewed	Online national survey	Ghana residents	National sample	Adherence to good handwashing practice
WASH	(UNICEF Ghana, 2024)	Official country page	Monitoring and programme data	Households in Ghana	Household handwashing place and materials	Availability of water and cleansing agent at home
WASH	(WHO/UNICEF JMP, 2021)	Official database	Household WASH indicators	Ghana households	National estimates by service level	Basic hygiene and safely managed water

Notes: i. Where a numeric sample is not listed in the source, we report the frame used (for example, DHIMS2 nationwide routine records or NHIS claims across 502 facilities). ii. Only Ghana-specific sources that underpin the results’ magnitudes are listed here. Global or regional studies are used in the Introduction and Discussion Sections for context and are not counted in this table.

**Table 2:** Number of Ghana-specific sources by domain and type, with a short note on sample coverage.

Domain	Ghana Specific Sources, Total	Peer Reviewed Empirical	Official or Administrative	Short Note on Sample Coverage
Mental health	1	1	0	District situation analysis of services and treatment gap
Gambling and risky behaviours	2	2	0	One rural adolescent survey; one national survey of 5024 children
Health system access	4	3	1	DHIMS2 nationwide doses; NHIS claims from 502 facilities; two district case settings; national DTP3
Economic hardship and child labour	2	0	2	National phone surveys tracking income and child work
Nutrition	3	1	2	School feeding reach; national food insecurity estimates; household monitoring
Education	4	0	4	National phone survey of modality use; national policy and implementation records
Early childhood development	2	2	0	Follow-up of ECE cohorts; qualitative study with preschool teachers and centres
WASH	3	1	2	National survey of handwashing practice; household service level estimates
Total (domain sum)	21	10	11	Domain counts include reuse of sources across domains

## Data Availability

No new data were created or analysed in this study. Data sharing is not applicable to this article.

## Abbreviations

The following abbreviations are used in this manuscript:

COVID-19	Coronavirus disease 2019
ANC	Antenatal care
DHIMS2	District Health Information Management System 2
DTP3	Third dose of diphtheria tetanus pertussis containing vaccine
ECD	Early childhood development
ECE	Early childhood education
FAO	Food and Agriculture Organization of the United Nations
FIES	Food Insecurity Experience Scale
GES	Ghana Education Service
GSS	Ghana Statistical Service
IFAD	International Fund for Agricultural Development
JMP	WHO and UNICEF Joint Monitoring Programme for Water Supply, Sanitation and Hygiene
KG	Kindergarten
LEAP	Livelihood Empowerment Against Poverty
LMIC(s)	Low- and middle-income country or countries
NHIS	National Health Insurance Scheme (Ghana)
OPD	Outpatient department
PNC	Postnatal care
SD	Standard deviation
SHS	Senior high school
SRH	Sexual and reproductive health
UNESCO	United Nations Educational Scientific and Cultural Organization
UNFPA	United Nations Population Fund
UNICEF	United Nations Children’s Fund
WHO	World Health Organization
WHO AFRO	World Health Organization Regional Office for Africa
WASH	Water sanitation and hygiene
